# Pictorial essay: Congenital anomalies of male urethra in children

**DOI:** 10.4103/0971-3026.76053

**Published:** 2011

**Authors:** Manisha Jana, Arun K Gupta, Kundum R Prasad, Sandeep Goel, Vishal D Tambade, Upasna Sinha

**Affiliations:** Department of Radiodiagnosis, All India Institute of Medical Sciences, Ansari Nagar, New Delhi, India

**Keywords:** Congenital urethral anomalies, intravenous urogram, voiding cystourethrogram

## Abstract

Congenital anomalies of the male urogenital tract are common. Some lesions like posterior urethral valve or anterior urethral diverticulum tend to present early in infancy and are often easily diagnosed on conventional contrast voiding cystourethrograms. Other conditions like posterior urethral diverticulum or utricle can be relatively asymptomatic and therefore present late in childhood. We present the spectrum of imaging findings of common and uncommon anomalies involving the male urethra. Since the pediatric radiologist is often the first to make the diagnosis, he or she should be well aware of these conditions.

## Introduction

Congenital anomalies of the urogenital tract are among the commonest anomalies found in the fetus, neonate, and infant.[1] Most of these anomalies can be easily diagnosed by conventional contrast voiding cystourethrogram (VCUG), retrograde urethrogram (RGU), intravenous urography (IVU), or nuclear imaging, and can be successfully treated with a good outcome. Hence, the radiologist should be aware of not just the common but also the uncommon congenital anomalies and their imaging correlates; this requires a good knowledge of the anatomy and embryology of the genitourinary tract.

Of the congenital anomalies of the urogenital tract, many involve the male urethra [Table 1], sometimes with associated anomalies of the external genitalia or anorectal malformations. This article attempts to give a pictorial overview of the congenital anomalies of the male urethra in children.

**Table 1:** Congenital anomalies involving the male urethra

Absent phallus/ agenesis of urethra[3]
Hypospadias
Epispadias-exstrophy complex
Congenital urethral duplication
-Partial duplication
-Complete duplication
-Duplication of urethra as a part of complete caudal duplication
Congenital urethral fistulae
-H type urethroperineal fistula
-Rectourethral fistula/ anourethral fistula with anorectal atresia
Posterior urethral valve
Anterior urethral valve
Diverticulae
-Posterior urethral diverticulum
-Anterior urethral diverticulum
Congenital megalourethra
-Scaphoid megalourethra
-Fusiform megalourethra
Prune- belly syndrome
Posterior urethral polyp
Prostatic utricle
Congenital meatal stenosis
Congenital urethral stenosis

## Embryology

The male urethra can be divided into a proximal pelvic urethra and a distal phallic urethra. The pelvic urethra (prostatic and membranous urethra) develops from the urogenital sinus. The phallic urethra (bulbar and penile part) develops from the degeneration of the urethral plate and ventral fusion of the urethral folds between the 8th and 12^th^ week of gestation.[2]

## Congenital Conditions

### Hypospadias

**Figure 1:**
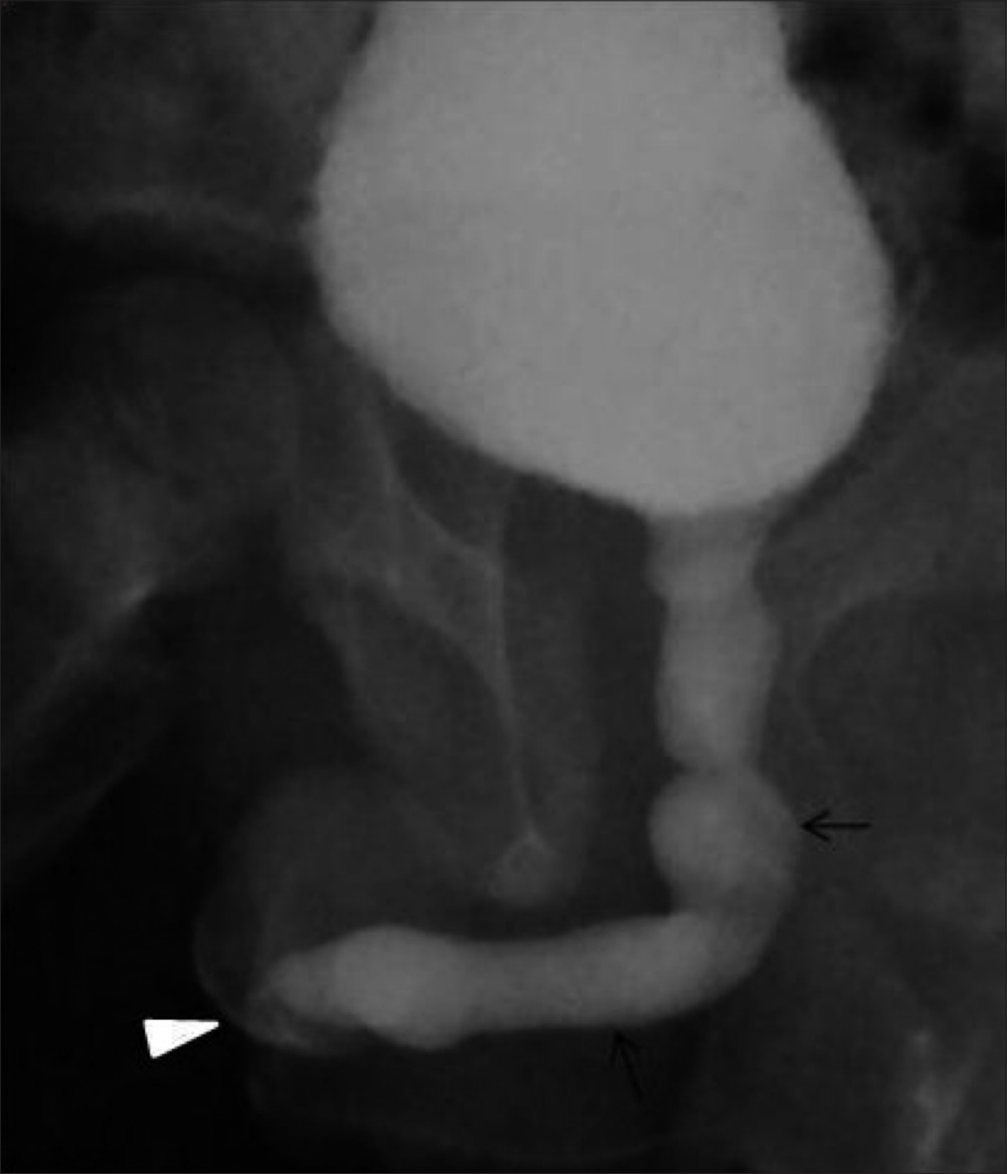
Penile hypospadias with meatal stenosis in a 10-year-old male presenting with a short phallus and thin urinary stream. An oblique VCUG image reveals a uniformly dilated urethra up to the tip (arrows) and abrupt narrowing of the urinary stream at the hypospadiac meatus (arrowhead)

Hypospadias is the most common congenital urethral anomaly. It is sometimes associated with other urogenital abnormalities. The urethral meatus is located on the ventral surface, anywhere from the penile shaft to the penoscrotal region, and is associated with a dorsal chordee [Figure 1].

### Epispadias

**Figure 2:**
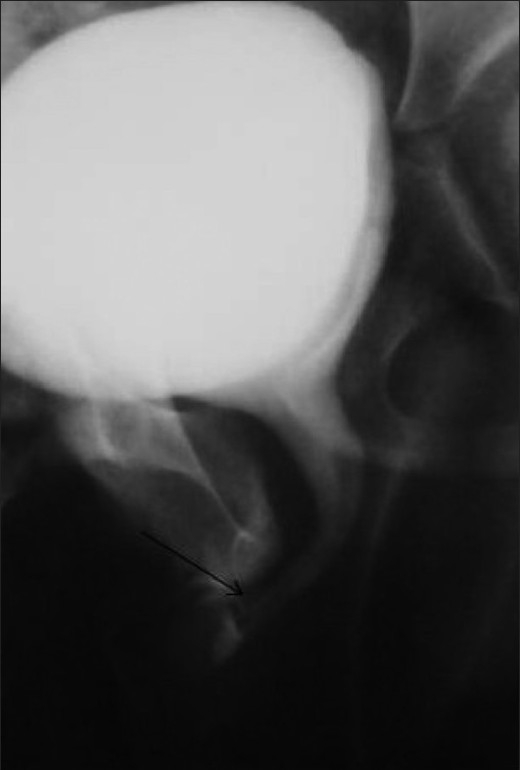
An oblique VCUG image of a young male shows a short epispadiac urethra opening on the dorsal surface of the penile shaft (arrow)

**Figure 3:**
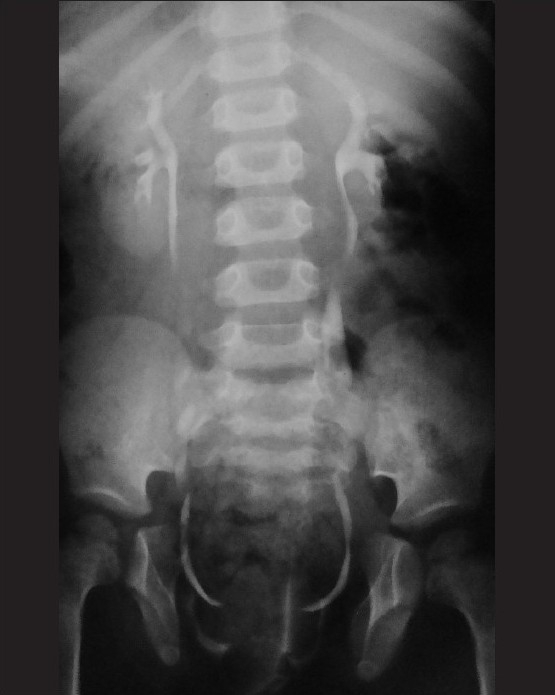
An IVU image of a child with bladder exstrophy shows pubic diastasis. Note the lack of a well-formed bladder (contrast from the ureters can be seen escaping out on to the skin surface)

Epispadias can be isolated or seen as part of the exstrophy-epispadias complex. The urethral meatus is located dorsally on the penile shaft [Figure 2]. In severe forms associated with exstrophy, there is a deficient lower anterior abdominal wall and anterior urinary bladder wall, a small phallus, and widely divergent pubic bones [Figure 3]. The ureters take an abnormal lateral and upward curvature at the terminal part to give a hooked or “Hurley-stick appearance.”[3]

### Congenital urethral duplication

**Figure 4(A,B):**
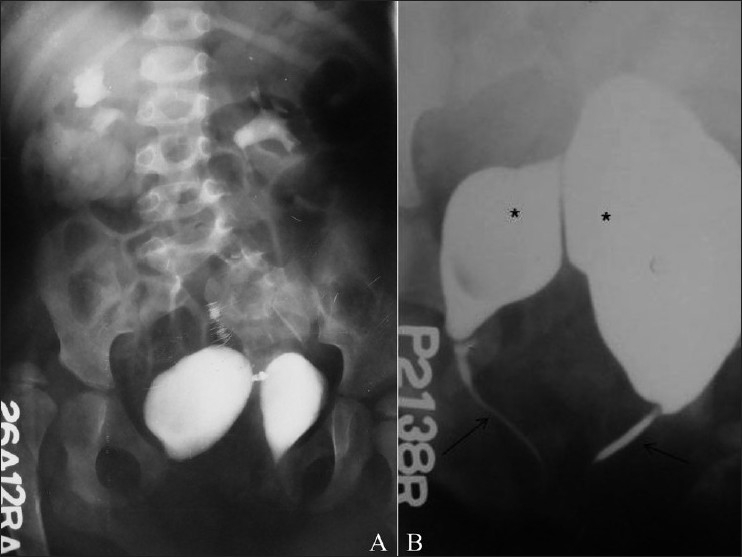
Complete caudal duplication. The IVU image (A) reveals the duplication of the urinary bladder. Note the pubic diastasis and developmental dysplasia involving the right hip joint. A VCUG image (B) shows complete duplication of the bladder (*) and urethra (arrows)

**Figure 5:**
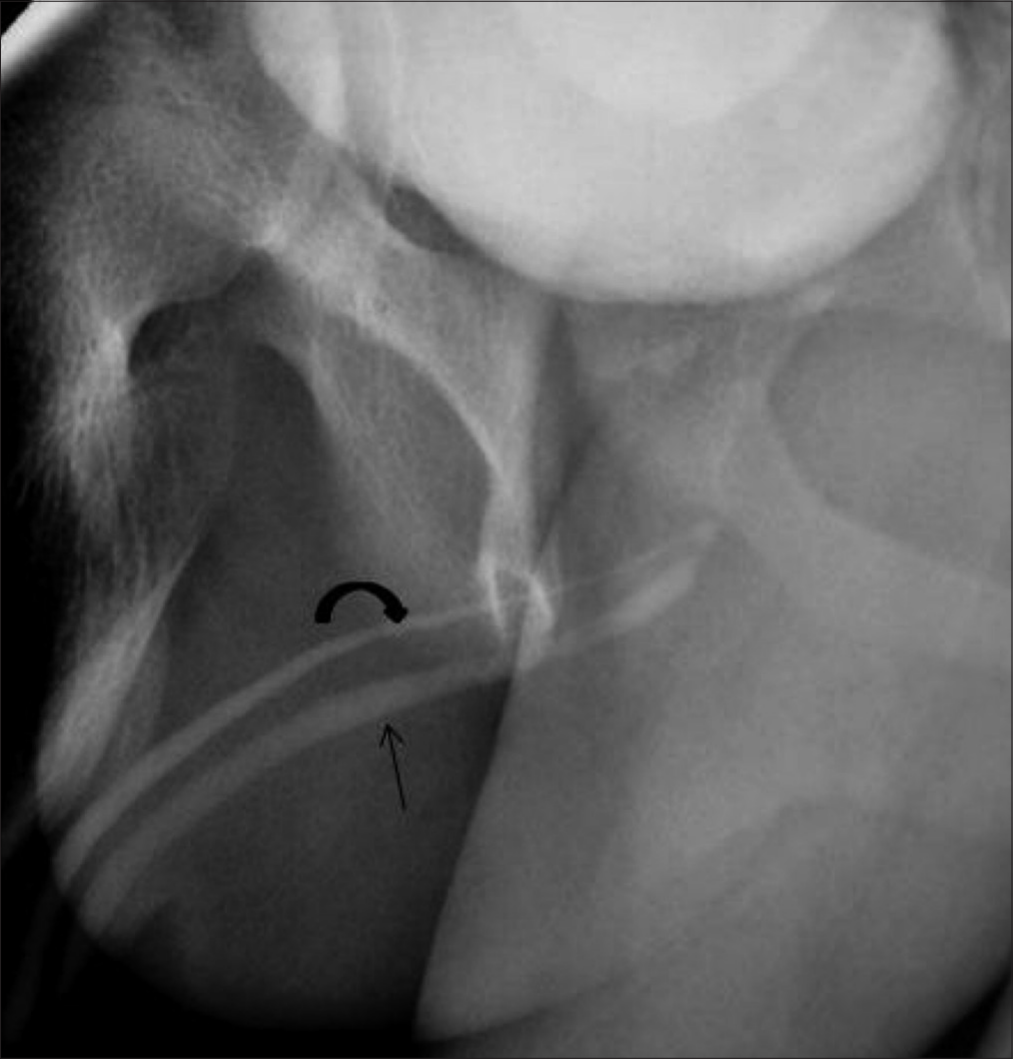
Hypospadiac type of partial urethral duplication (Effmann type IIA2) in a 12-year-old boy with a history of a double urinary stream. An oblique VCUG image reveals two separate meati, two incomplete urethral channels joining at the posterior urethra. The ventral hypospadiac channel is of normal caliber (arrow), whereas the orthotopic dorsal one has a small caliber (curved arrow)

**Figure 6:**
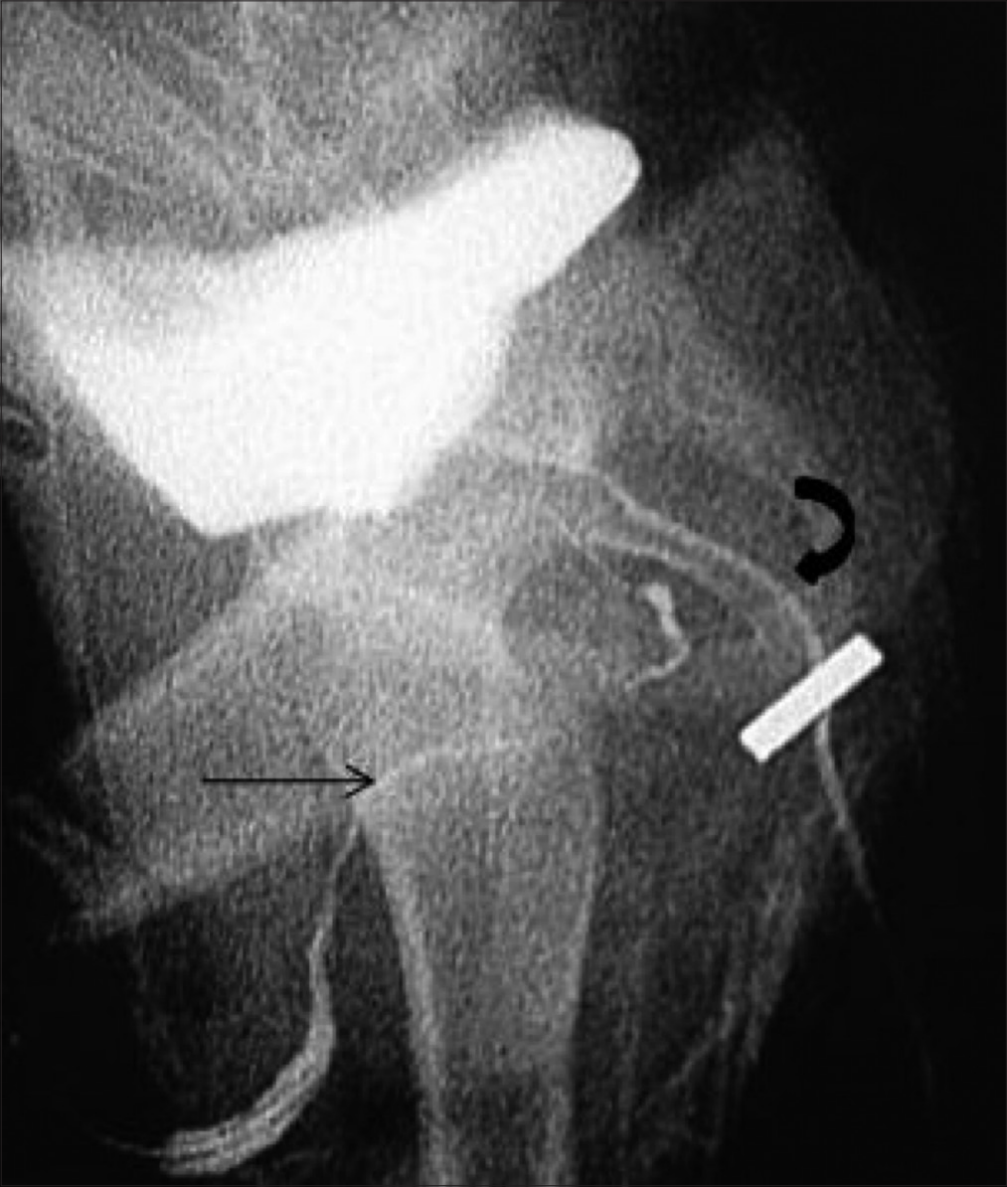
Hypospadiac type of complete urethral duplication (Effmann type IIA1) in a child presenting with passage of urine through a preanal opening and absence of micturition through the normal urethral meatus. An oblique RGU image performed from both the meati reveals a dorsal orthotopic channel of narrow calibre (arrow), with a ventral channel (curved arrow) originating separately from the bladder

**Figure 7:**
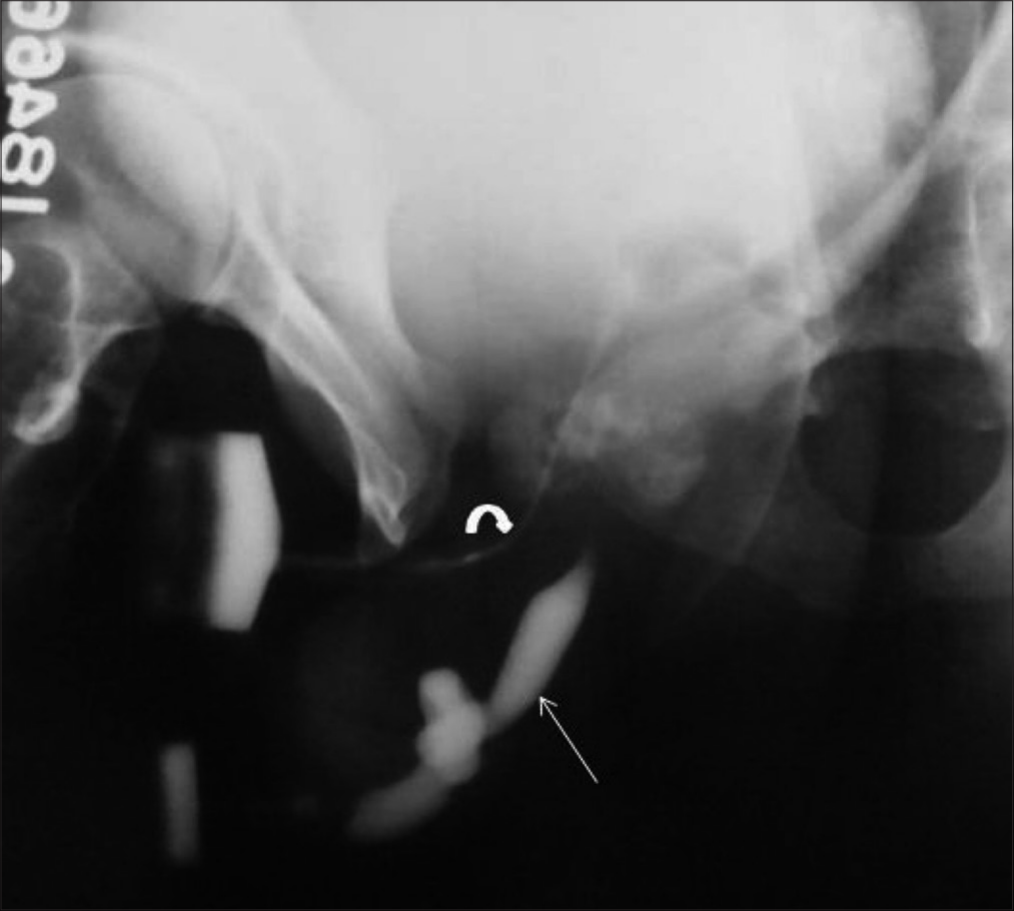
Epispadiac type of complete urethral duplication in a child with a double urinary stream. An oblique RGU image (performed after injecting iodinated contrast simultaneously from both the orifices) shows complete urethral duplication. The ventral orthotopic channel is dilated (arrow), whereas the dorsal ectopic channel is narrow in calibre (curved arrow)

Urethral duplication can be divided into the following types:[4,5] type I, blind and incomplete; type IIA, complete patent duplication, with two meati; type IIB, complete patent duplication, with both the urethrae joining distally and opening through a single meatus; and type III, urethral duplication occurring as part of a very rare anomaly termed complete caudal duplication.[6] In complete caudal duplication, the bladder is usually completely divided in the sagittal plane, each half receiving one (ipsilateral) ureter and having a separate urethra [Figure 4A–B]. Rarely, there may be a single urethra leading to outlet obstruction of one of the bladders.[7,8] Urethral duplication commonly occurs in the sagittal plane, though rare cases of duplication in the coronal plane have also been reported.[9] Duplication of the urethra could be partial [Figure 5] or complete [Figure 6] and either hypospadiac or epispadiac [Figure 7], depending on the relation of the accessory channel with the orthotopic urethra. In the rare epispadiac type, there is a dorsal accessory urethral opening and the child is usually incontinent.

### H- or N-type recto- or anoprostatic urethral fistula

This is an extremely rare type of anorectal malformation. There is a fistulous communication between the prostatic urethra and the anterior wall of the rectum or anus. Typically, the urethra distal to the site of the fistula is narrow and stenotic, resulting in a poor urinary stream.[10] Some studies suggest that the ventral urethra is usually functional in all cases of hypospadiac urethral duplication, whereas in congenital urethroperineal fistula the dominant urinary stream is through the dorsal orthotopic channel.[11]

### Rectourethral fistula associated with anorectal malformation

**Figure 8:**
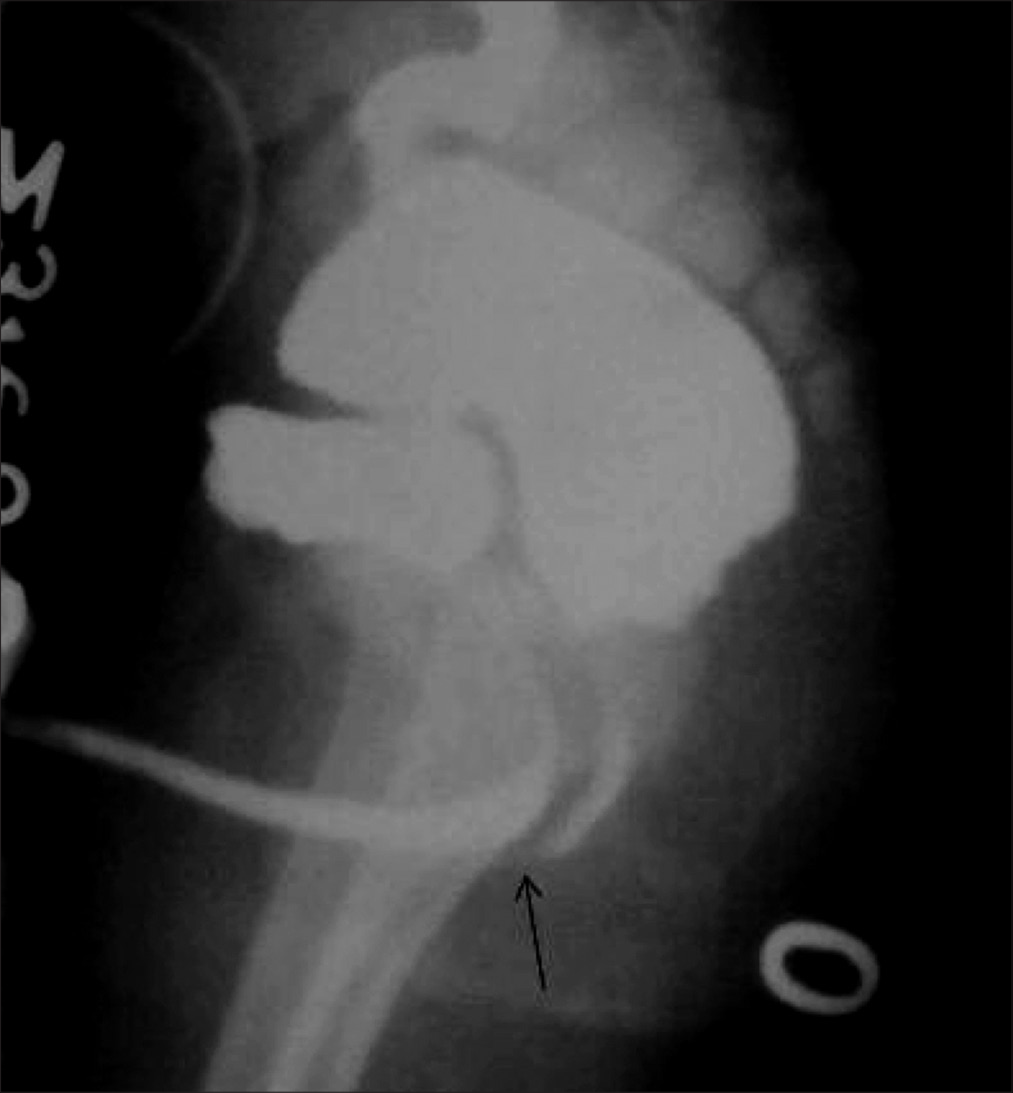
Rectobulbar fistula with anorectal atresia in an infant who presented in the early neonatal period with passage of meconium through the urethral route. A distal cologram (performed through a transverse colostomy) reveals the absence of the distal rectum and anal canal, with communication between the rectum and the bulbar urethra through a fistula (arrow)

**Figure 9:**
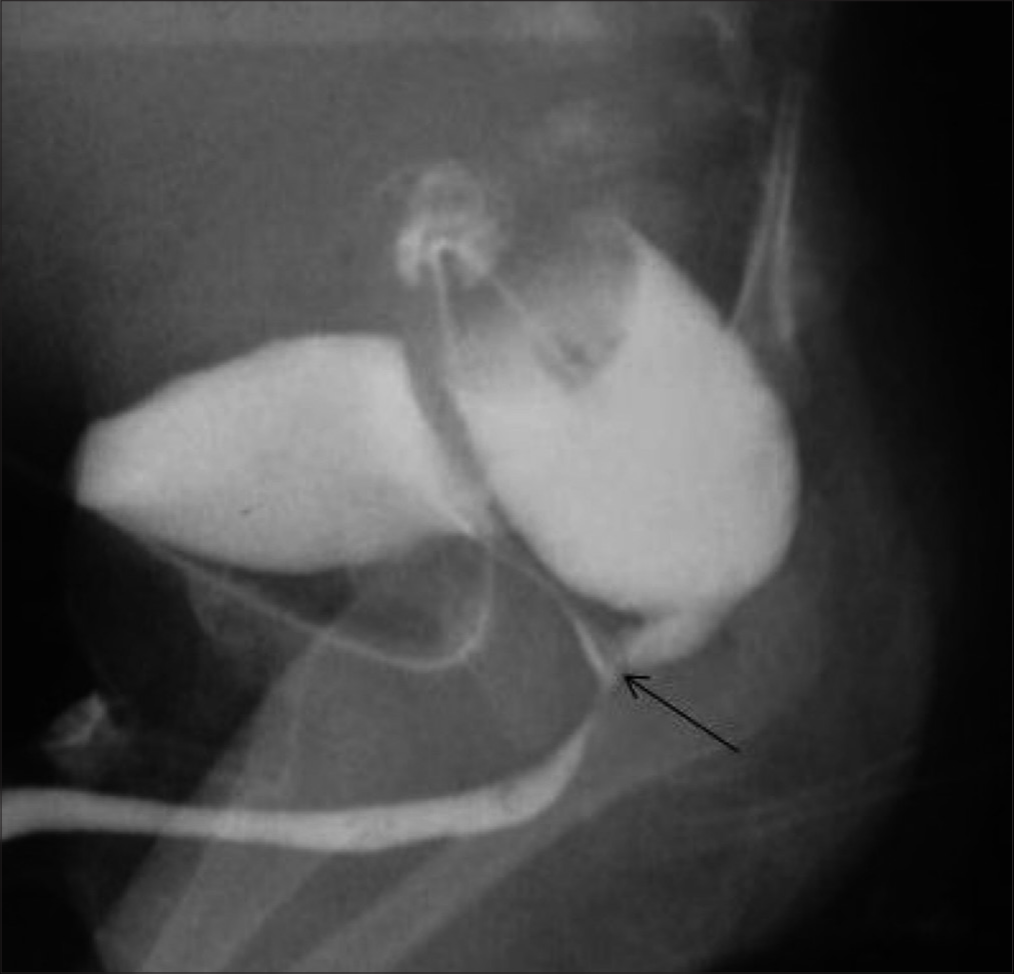
Rectobulbar fistula with anorectal agenesis in an 11-month-old male child who had no anal opening and a history of urethral passage of meconium. A contrast cologram performed through a sigmoid colostomy revealed a fistulous communication between the rectum and the bulbar urethra (arrow), along with agenesis of the distal rectum and anal canal

Congenital rectourethral fistula is usually associated with the high and intermediate type of anorectal malformations. A contrast study, either through the colostomy or via retrograde urethrography, demonstrates the fistulous tract in most patients. The fistulous communication is between the blind-ending rectum and either the bulbar urethra [Figures 8 and 9] or, more commonly, the prostatic urethra.[10,12]

### Posterior urethral valve

**Figure 10:**
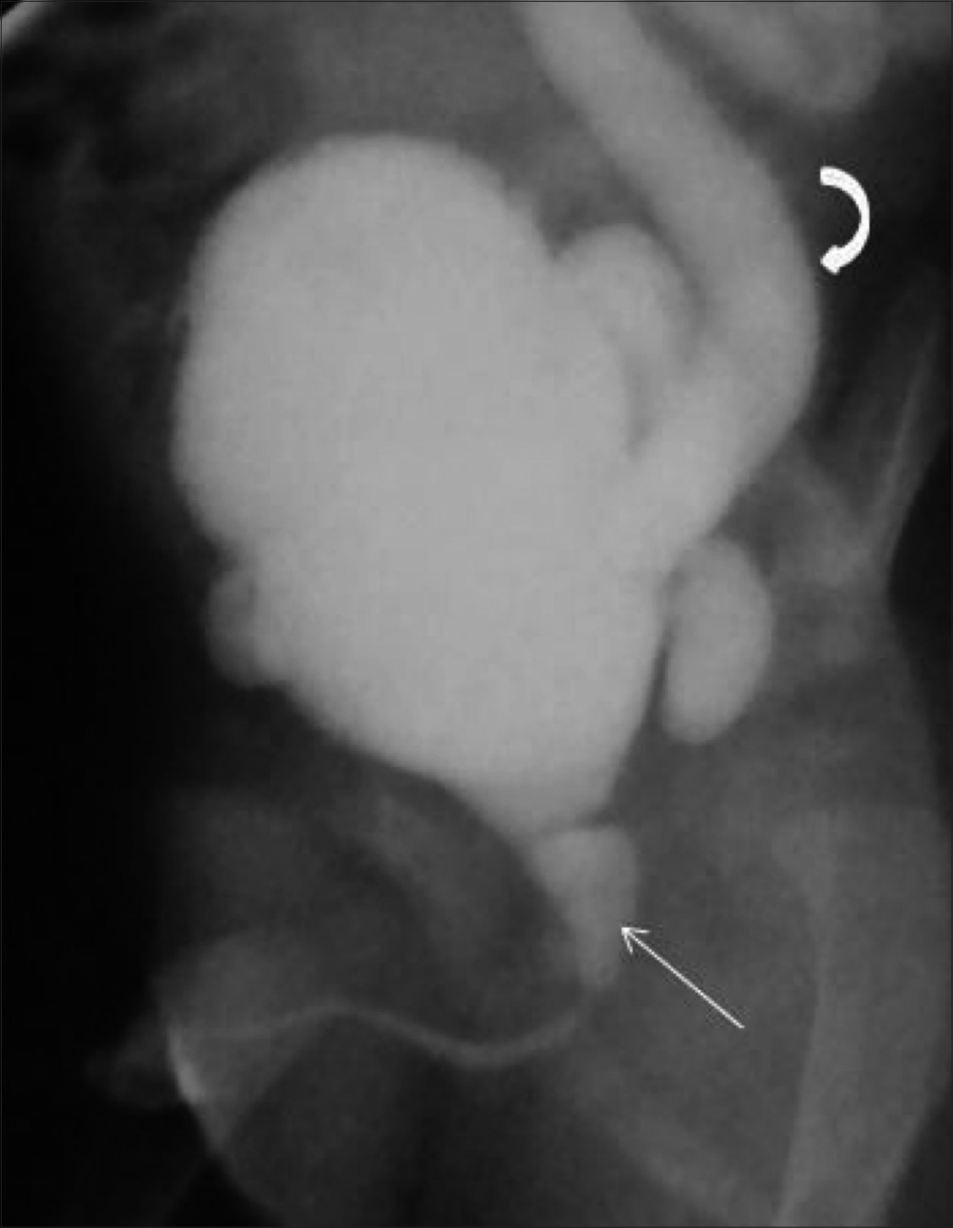
Posterior urethral valve in a 7-year-old male child. An oblique VCUG image shows a dilated posterior urethra (arrow) with an abrupt transition to a normal-calibre anterior urethra. Note the bladder neck hypertrophy, the irregular trabeculated bladder wall, and the left-sided grade III vesicoureteric reflux (curved arrow)

**Figure 11:**
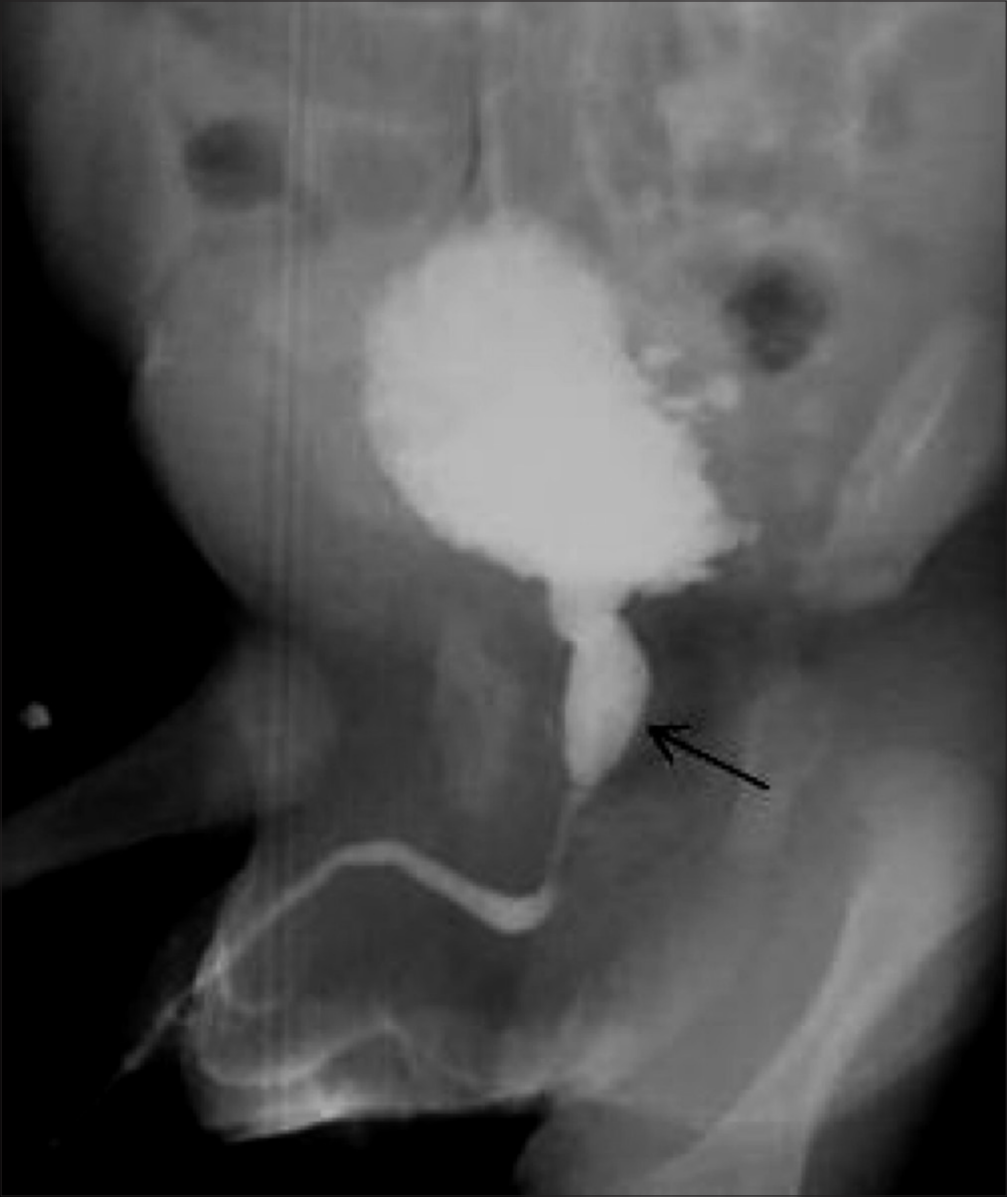
Posterior urethral valve in a newborn. An oblique VCUG image shows a dilated posterior urethra (arrow) and a trabeculated urinary bladder

Posterior urethral valves (PUVs) are the commonest cause of bladder outlet obstruction in a male child.[2] This condition may be diagnosed antenatally, in the neonatal period, or later, with the age at presentation depending on the degree of obstruction. Though earlier divided into three types,[13] currently only one type (formerly called type I) is recognized. PUVs can only be diagnosed with a VCUG and not with retrograde urethrography. VCUG shows a disproportionately dilated posterior urethra, with an abrupt transition into a narrow anterior urethra, bladder neck hypertrophy, and trabeculation/sacculation of the bladder [Figures 10 and 11], usually with a small capacity; also, there may or may not be associated vesicoureteric reflux. In high-grade obstruction, a neonate may present with perirenal urinoma, dysplastic kidneys, or urinary ascites.[14,15] Nowadays, the entity is termed congenital obstructive posterior urethral membrane (COPUM).

### Posterior urethral polyp

Posterior urethral polyp, a rare cause of intermittent urethral obstruction,[16] is an elongated pedunculated polypoid lesion attached to the verumontanum. On VCUG, the lesion appears as a lucent filling defect that moves downwards during micturition.

### Prostatic utricle

**Figure 12:**
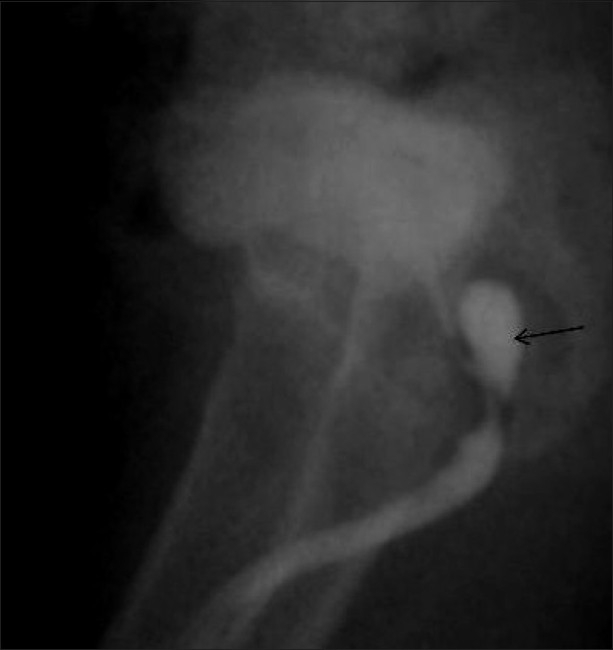
Prostatic utricle. Oblique RGU image reveals a blind-ending outpouching, filled with the contrast, arising from the prostatic urethra (arrow). The anterior urethra appears normal

The prostatic utricle is a small, blind-ending midline pouch arising from the prostatic urethra at the level of the verumontanum [Figure 12]. It represents the remnant of the caudal end of the fused Müllerian ducts.[17] A large prostatic utricle may be associated with urinary retention, stasis, and infection. It can be associated with hypospadias or the prune belly syndrome.[18]

### Posterior urethral diverticulum

**Figure 13:**
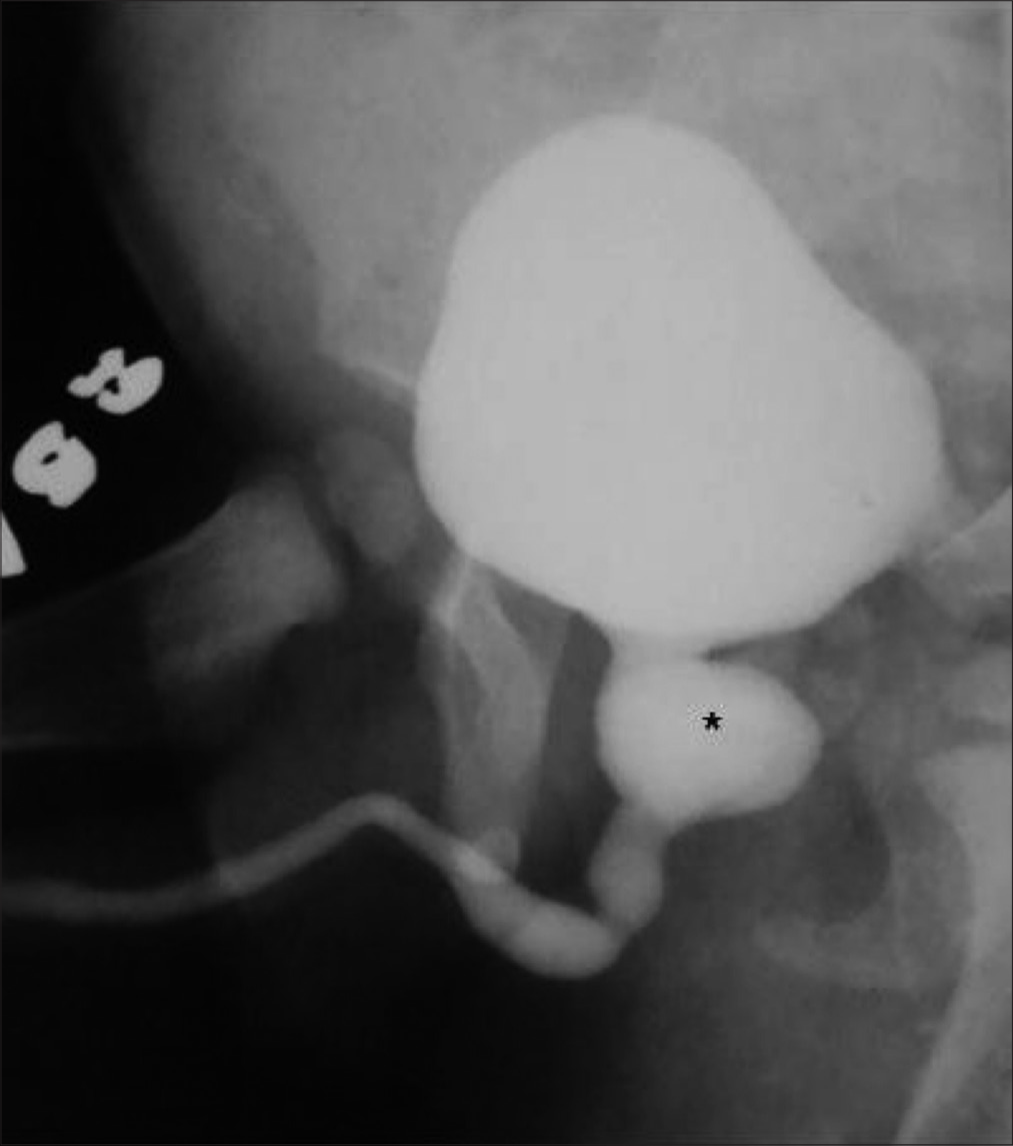
Large posterior urethral diverticulum in an 8-year-old male child presenting with recurrent urinary tract infection. An oblique VCUG image reveals a large wide-neck diverticulum (*) arising from the prostatic urethra

Most posterior urethral diverticulae are acquired in origin and lined with columnar epithelium or granulation tissue. A congenital posterior urethral diverticulum is a rare entity [Figure 13]. A large diverticulum may be complicated by urinary stasis, infection, and calculi formation.

### Anterior urethral diverticulum

**Figure 14:**
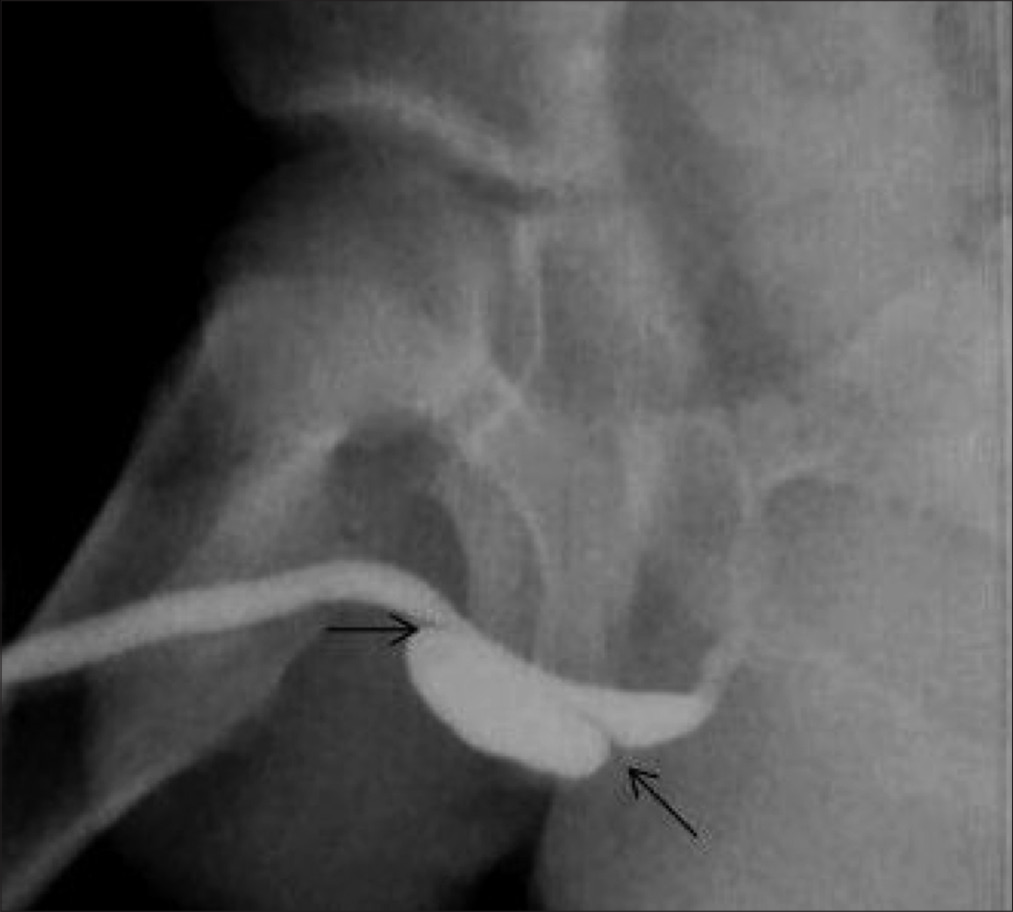
Anterior urethral diverticulum in a 5-year-old male child. An oblique VCUG image reveals a large diverticulum arising from the ventral surface of the penile urethra. Note the prominent anterior as well as posterior lips (arrows). The diverticulum caused external compression of the penile urethra, leading to a poor urinary stream

**Figure 15:**
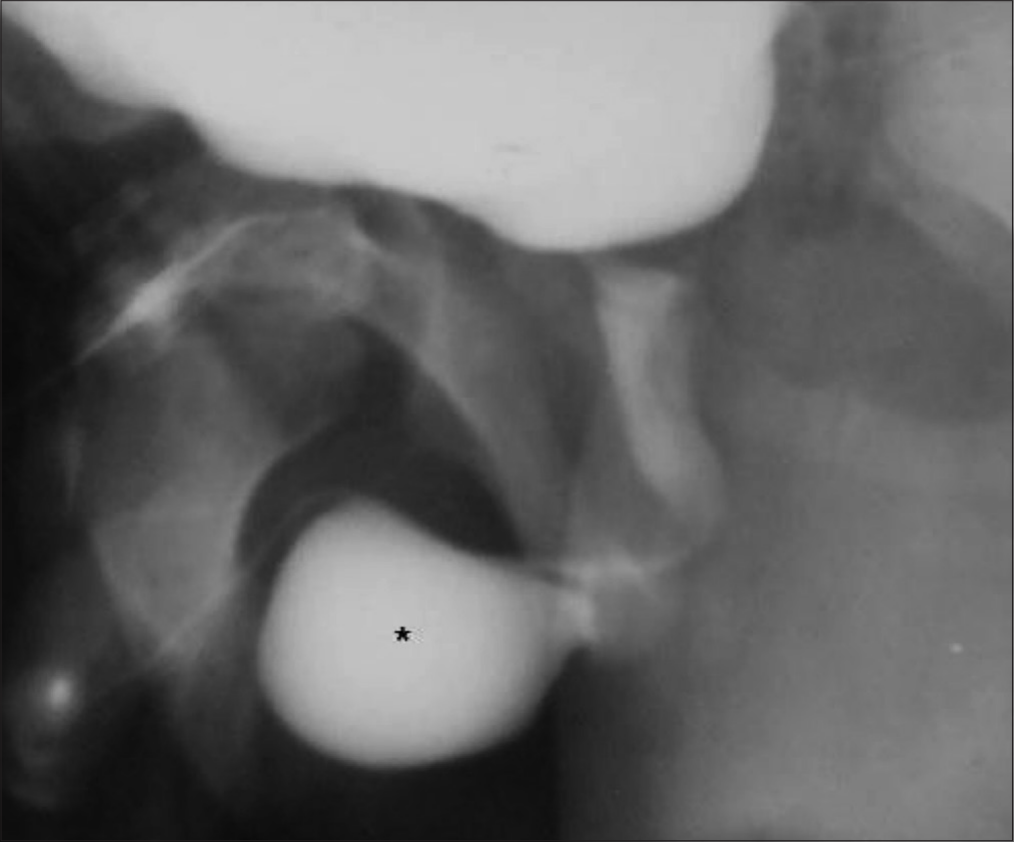
A huge anterior urethral diverticulum arising from the bulbar urethra in a 10-year-old male child. The boy had a history of a swelling at the penoscrotal region during micturition. An oblique VCUG image reveals a large ventral diverticulum (*) with a narrow neck

**Figure 16:**
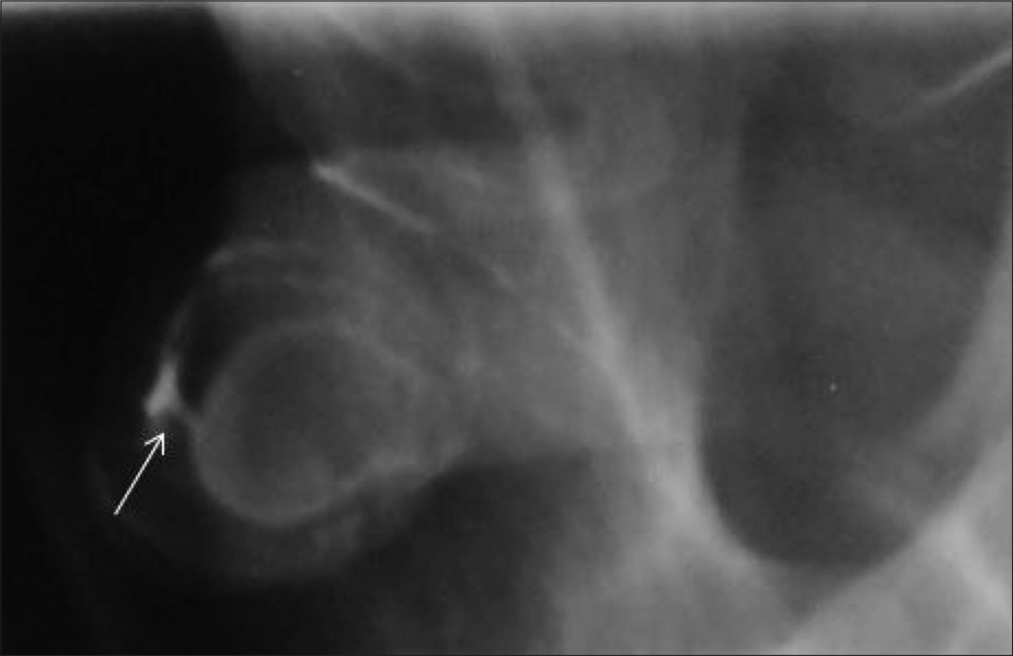
Anterior urethral diverticulum arising from the distal penile urethra and having a very narrow neck. A double-contrast RGU (using air and iodinated contrast agent) image provides an excellent delineation of the diverticulum and its communication with the penile urethra (arrow)

An anterior urethral diverticulum is a saccular outpouching arising from the ventral surface of the anterior urethra. Two types are described. Most commonly it arises from the ventral surface of the bulbar urethra [Figures 14 and 15]. The other rarer type is found located near the penile tip [Figure 16] and has a short neck. The former usually presents with obstruction[19–21] to the urinary stream, while the latter is more prone to calculus formation.

### Anterior urethral valve

An anterior urethral valve is a posteriorly directed semilunar fold arising from the floor of the anterior urethra and causing urethral obstruction during micturition. On imaging, it can mimic an anterior urethral diverticulum, but the posterior lip is absent in a valve.

### Congenital megalourethra

**Figure 17:**
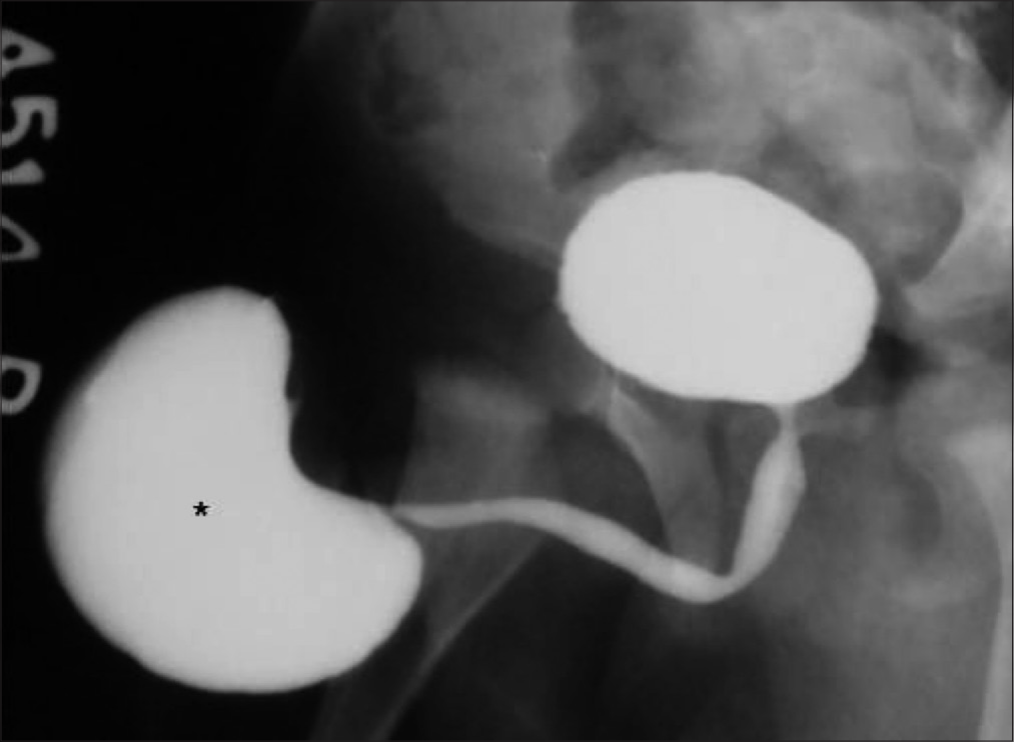
Scaphoid megalourethra in an infant with a history of a huge swelling at the penile tip during micturition. On pressing the swelling between the fingers, urine could be squeezed out. An oblique VCUG image reveals a huge scaphoid, contrast-filled structure at the distal penile urethra (*); it is more prominent ventrally. The posterior urethra and bulbar urethra are normal

**Figure 18:**
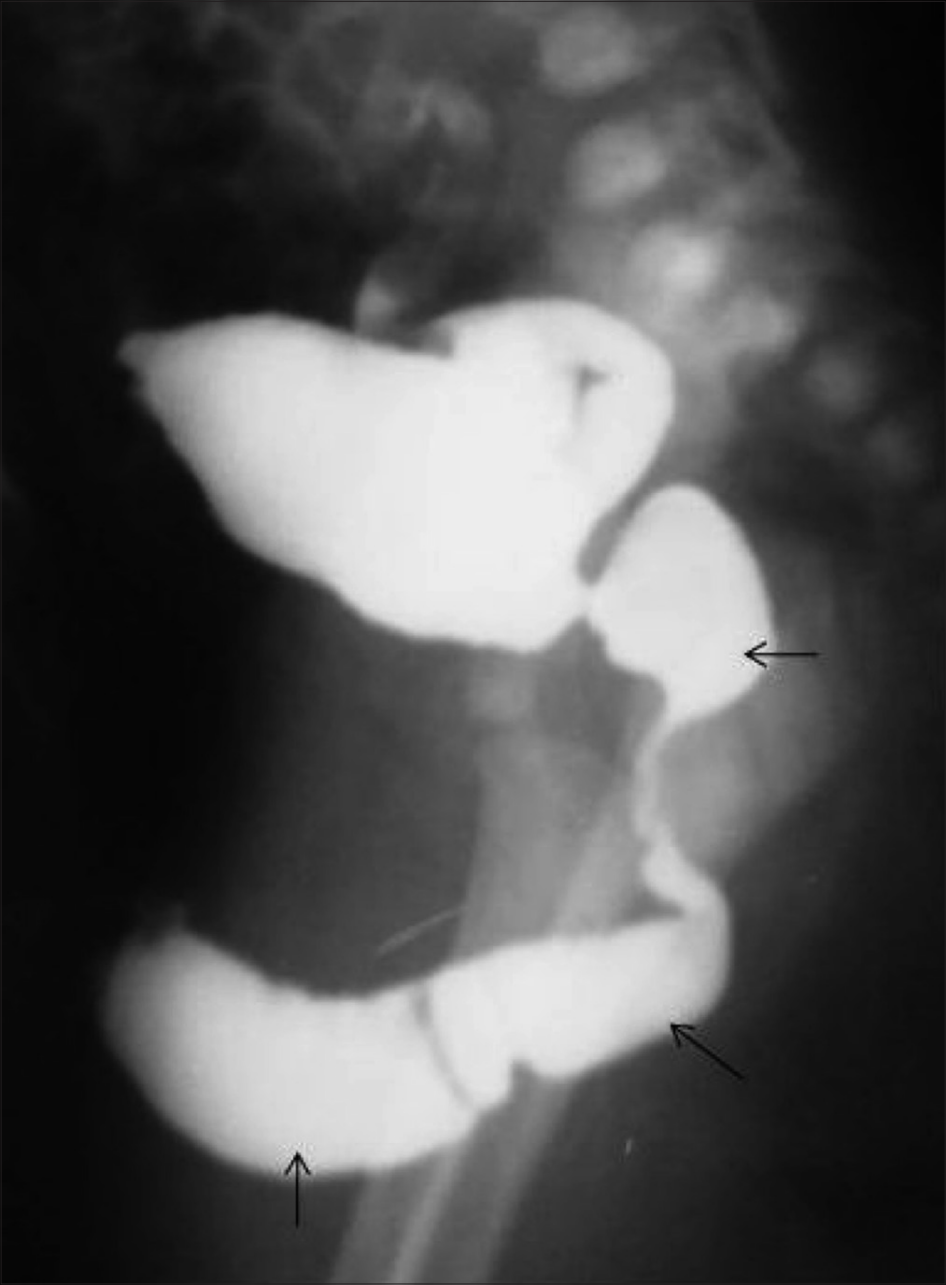
Fusiform megalourethra in an infant. Lateral VCUG image reveals an extensively dilated anterior and posterior urethra (arrows)

This is a rare congenital anomaly resulting from the faulty development of the corpora cavernosa and corpus spongiosum. Two types are described. The milder and commoner form, scaphoid megalourethra, results from a localized underdevelopment or deficiency of the corpus spongiosum [Figure 17], [22,23] with intact corpora cavernosa. Fusiform megalourethra is the rarer, more severe, form in which there is deficiency of the corpora cavernosa as well as the corpus spongiosum,[22,24] resulting in diffuse dilatation of the penile urethra [Figure 18].

### Prune belly syndrome

**Figure 19(A-C):**
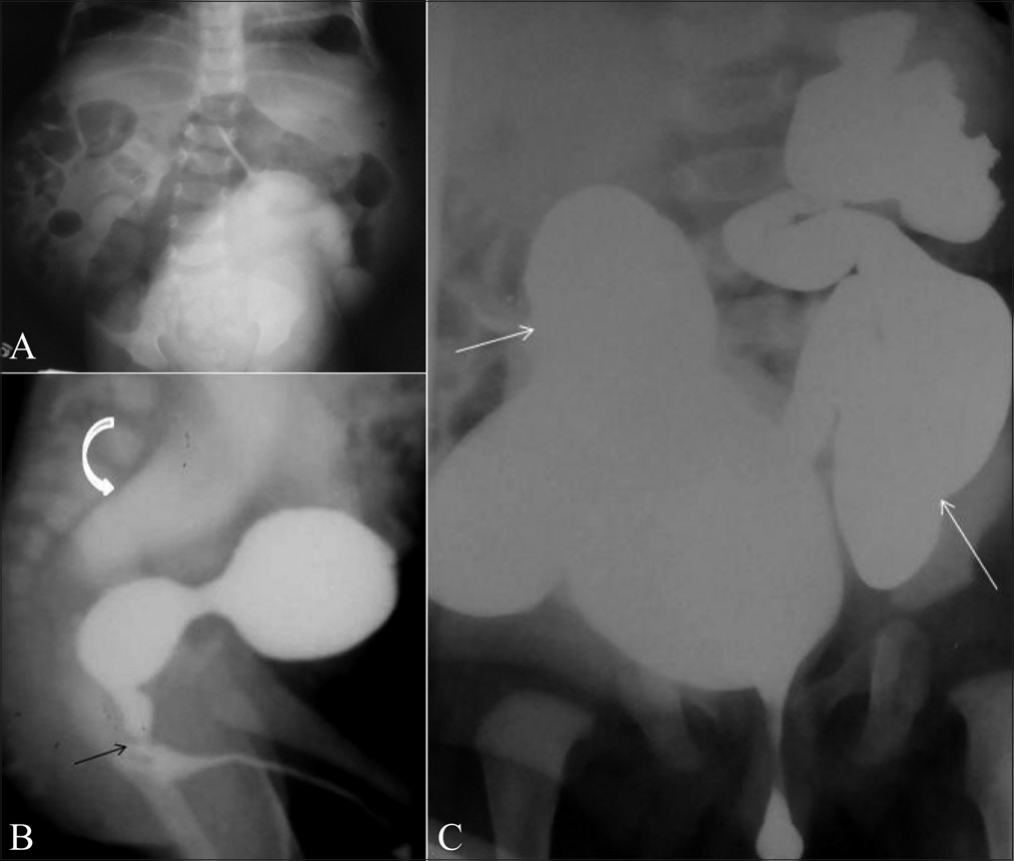
Prune belly syndrome in a newborn. The infant had a lax and winkled anterior abdominal wall and the scrotal sacs were empty. A frontal abdominal radiograph (delayed postcontrast image, A) reveals the lax and patulous abdominal wall manifested by the distended flanks. An oblique VCUG image (B) reveals an elongated, dilated, and patulous posterior urethra, with tapered transition into a normal anterior urethra (arrow). Note the grossly dilated ureters and the vesicoureteric reflux (curved arrow). It is the tapered transition of the posterior urethra that helps differentiate this condition from PUV, in which there is an abrupt transition. There is bilateral grade V vesicoureteric reflux (arrow) and grossly tortuous laterally placed ureters (C), in this frontal VCUG image

This refers to a constellation of anomalies, including lax abdominal wall musculature, cryptorchidism, and various lower urinary tract anomalies.[25–27] The posterior urethra is typically dilated, high-placed, and tapered distally; the appearance may mimic a posterior urethral valve [Figure 19A–C].

### Congenital meatal stenosis

Congenital meatal stenosis is most frequently associated with hypospadias. On VCUG, the entire urethra up to the meatus is dilated [Figure 1].

## Conclusion

A number of congenital conditions can affect the male urethra, and the diagnosis is predominantly based on VCUG, RGU, and USG. Congenital causes of urethral obstruction like PUV can be diagnosed on antenatal USG or MRI. Though VCUG may be essential for diagnosis, radiation issues should be taken into consideration when performing the investigation in a newborn or an infant.
